# Patient enablement and health-related quality of life for patients with chronic back and knee pain: a cross-sectional study in primary care

**DOI:** 10.3399/BJGP.2022.0546

**Published:** 2023-10-17

**Authors:** Amy Pui Pui Ng, John King Yiu Cheng, Joyce Sau Mei Lam, Carlos King Ho Wong, Will Ho Gi Cheng, Emily Tsui Yee Tse, David Vai Kiong Chao, Edmond Pui Hang Choi, Rosa Sze Man Wong, Cindy Lo Kuen Lam

**Affiliations:** Department of Family Medicine, University of Hong Kong-Shenzhen Hospital, Shenzhen, China; Department of Family Medicine and Primary Care, School of Clinical Medicine, Li Ka Shing Faculty of Medicine, University of Hong Kong, Hong Kong, China.; Department of Family Medicine and Primary Care, School of Clinical Medicine, Li Ka Shing Faculty of Medicine, University of Hong Kong, Hong Kong, China.; Department of Family Medicine and Primary Care, School of Clinical Medicine, Li Ka Shing Faculty of Medicine, University of Hong Kong, Hong Kong, China.; Department of Family Medicine and Primary Care, School of Clinical Medicine, Li Ka Shing Faculty of Medicine, University of Hong Kong, Hong Kong; Department of Pharmacology and Pharmacy, Li Ka Shing Faculty of Medicine, University of Hong Kong, Hong Kong; Laboratory of Data Discovery for Health (D^2^4H) Hong Kong Science and Technology Park, Sha Tin, Hong Kong SAR, China.; Department of Family Medicine and Primary Care, School of Clinical Medicine, Li Ka Shing Faculty of Medicine, University of Hong Kong, Hong Kong, China.; Department of Family Medicine, University of Hong Kong-Shenzhen Hospital, Shenzhen, China; Department of Family Medicine and Primary Care, School of Clinical Medicine, Li Ka Shing Faculty of Medicine, University of Hong Kong, Hong Kong, China.; Department of Family Medicine & Primary Health Care, Kowloon East Cluster, Hospital Authority, Hong Kong SAR, China.; School of Nursing, Li Ka Shing Faculty of Medicine, University of Hong Kong, Hong Kong, China.; Department of Pharmacology and Pharmacy, Li Ka Shing Faculty of Medicine, University of Hong Kong, Hong Kong, China.; Department of Family Medicine, University of Hong Kong-Shenzhen Hospital, Shenzhen, China; Department of Family Medicine and Primary Care, School of Clinical Medicine, Li Ka Shing Faculty of Medicine, University of Hong Kong, Hong Kong, China.

**Keywords:** back pain, chronic pain, knee, health-related quality of life, musculoskeletal pain, primary care

## Abstract

**Background:**

Chronic back and knee pain impairs health- related quality of life (HRQoL) and patient enablement can improve HRQoL.

**Aim:**

To determine whether enablement was a moderator of the effect of chronic back and knee pain on HRQoL.

**Design and setting:**

A cross-sectional study of Chinese patients with chronic back and knee problems in public primary care clinics in Hong Kong.

**Method:**

Each participant completed the Chinese Patient Enablement Instrument-2 (PEI-2), the Chinese Western Ontario and McMaster Universities Osteoarthritis Index (WOMAC), and the Pain Rating Scale (PRS). Multivariable regression examined the effects of PRS score and PEI-2 score on WOMAC total score. A moderation regression model and simple slope analysis were used to evaluate whether the interaction between enablement (PEI-2) and pain (PRS) had a significant effect on HRQoL (WOMAC).

**Results:**

Valid patient-reported outcome data from 1306 participants were analysed. PRS score was associated with WOMAC total score (β = 0.326, *P*<0.001), whereas PEI-2 score was associated inversely with WOMAC total score (β = −0.260, *P*<0.001) and PRS score. The effect of the interaction between PRS and PEI-2 (PRS × PEI-2) scores on WOMAC total score was significant (β = −0.191, *P*<0.001) suggesting PEI-2 was a moderator. Simple slope analyses showed that the relationship between PRS and WOMAC was stronger for participants with a low level of PEI-2 (gradient 3.056) than for those with a high level of PEI-2 (gradient 1.746).

**Conclusion:**

Patient enablement moderated the impact of pain on HRQoL. A higher level of enablement can lessen impairment in HRQoL associated with chronic back and knee pain.

## INTRODUCTION

Musculoskeletal problems not only diminish functioning, increase distress, and worsen self-perceived health for individuals, but also constitute a burden on the healthcare system.^1^^–^4 They account for 7% of primary care consultations in Hong Kong, with back and knee problems being the most prevalent.^5^

Health-related quality of life (HRQoL) is a patient-reported outcome (PRO) that assesses a person’s subjective judgement on how their health has an impact on their life.^6^ It can inform decisions on service needs and outcomes.^7^ Chronic musculoskeletal pain impairs HRQoL,^8^^,^^9^ disturbs sleep, and induces psychological distress.^10^^,^^11^

Patient enablement is defined as *‘the extent to which a patient is capable of understanding and coping with his or her health issues’*^12^ and is an indicator of the quality of consultations.^13^^–^15 It starts with a better understanding of patients’ needs and expectations, and encompasses shared decision making with acknowledgement of patients’ strengths^16^^–^18 by providing them with the ability to look after their illness.^17^^,^^19^ Patients with better enablement are more likely to have better chronic disease outcomes.^20^ Furthermore, patient enablement is modifiable and is a goal of patient-centred care.^20^ Tracking PROs, such as patient enablement, can help to assess the effectiveness of patient-centred care programmes.^21^ There are two major components of patient enablement — health literacy^22^ and coping.^23^ Health literacy is defined by Liu *et al* as the *‘ability of an individual to obtain and translate knowledge and information … to maintain and improve health in a way that is appropriate to the individual and system contexts’*.^24^^,^^25^ Coping is defined as *‘effortful behaviour undertaken in reaction to a stressor’*,^26^ which moderates the association between pain and HRQoL.^27^ Thus, more enabled individuals may maintain better HRQoL than those who are less enabled for the same level of pain.^28^

**Table:** How this fits in

Previous research shows that pain can negatively affect health-related quality of life (HRQoL) and that patient enablement, which is modifiable, can enhance HRQoL. This study showed that high levels of enablement can lessen the impairment of HRQoL related to chronic back and knee pain. Thus, the management of these patients should include patient enablement through education about their musculoskeletal problem to enhance health literacy, and promote self-management to cope with pain and exercise programmes to improve function.

There are other concepts intertwined with patient enablement. Illness perception focuses on how a patient experiences and mentally frames their life with illness,^29^ which affects coping strategies and self- management (that is, patient enablement)^30^ and HRQoL.^31^ The concepts of enablement and empowerment are sometimes used synonymously;^32^ however, patient enablement focuses on obtaining skills and knowledge, and patient empowerment involves gaining of power after obtaining those skills and knowledge, and, therefore, patient enablement must occur before patient empowerment.^33^

This study aimed to determine whether enablement was a moderator of the effect of chronic back and knee pain on HRQoL. The authors hypothesised that pain and enablement would affect HRQoL, and that the effect of pain on HRQoL could be moderated by enablement. The conceptual model is shown in Supplementary Figure S1.

## METHOD

### Study setting

The study was carried out in Hong Kong where the healthcare system is serviced by both the public and private sectors, and residents can freely choose where they receive care without means testing. As a result of the heavy financial subsidies, the public primary care clinics, called general outpatient clinics (GOPCs), are the main care providers for patients with chronic diseases.^34^ There are 73 GOPCs organised in seven clusters around Hong Kong. The care in GOPCs is standardised by evidence- based guidelines whereas care is more variable in the private sector. Participants of this study were recruited from six GOPCs in two clusters.

### Study design and participant recruitment

This cross-sectional study was part of a single-blinded cluster randomised controlled trial to evaluate the effectiveness of measuring and reporting HRQoL in routine clinical practice.^35^ Participant inclusion criteria were adults aged ≥18 years, who had a doctor-diagnosed symptomatic back and/or knee problem that was expected to last for ≥1 month. The exclusion criteria were patients who had a life expectancy <12 months, had current cancers, were too ill, or were unable to communicate in Chinese. All eligible patients were recruited by their doctors or trained research assistants in the clinics between 1 June 2020 and 31 December 2021. After completion of a written consent form, participants’ sociodemographic and clinical data were collected by structured questionnaires and PROs by a telephone survey administered by an independent survey organisation. The participant flow chart is shown in Supplementary Figure S2. More details on participant recruitment and data collection are described in another article.^35^

#### Sample size

A total of 1221 participants who had complete PRO data were included in this moderation study. Post hoc power analysis, using G*Power version 3.1.9.7,^36^ showed a power of 0.95 for moderation analysis at a significance criteria of α = 0.05, *F^2^* = 0.011.

### Study instruments

#### Structured questionnaires

Participants’ sociodemographic information, lifestyle factors, and self-reported comorbidities were collected by a questionnaire administered by a trained research assistant in the clinic. Another questionnaire was completed by the physician to collect data on the diagnosis and duration of musculoskeletal problems and to provide a global rating about the severity of the participant’s condition on a five-point Likert scale (GRS) from 1 (no problem) to 5 (very severe).

#### The Chinese Patient Enablement Instrument-2 (PEI-2)

The Chinese PEI-2 is a PRO measure that was adapted from the PEI originally developed by Howie *et al*^15^ and consists of six items: ability to cope with life; ability to understand one’s illness; ability to cope with one’s illness; ability to keep oneself healthy; confidence about one’s health; and ability to help oneself. Each item is rated on a 5-point Likert scale, ranging from 1 (not at all) to 5 (extremely well). The item scores are summated to give a total PEI-2 score (range: 6–30), with higher scores indicating better enablement. Missing item values can be imputed with the average of the scores of answered items, up to three items. The Chinese PEI-2 is valid, reliable, and sensitive in a Chinese population.^14^

#### The Chinese Western Ontario and McMaster Universities Osteoarthritis Index (WOMAC)

The WOMAC is a widely used HRQoL measure specific to musculoskeletal problems, which has been applied to patients with knee^37^ and back^38^ problems. It consists of 24 items in three domains: pain (five items); stiffness (two items); and physical function (17 items). Each item is rated on a 5-point Likert scale (0 to 4), with higher scores indicating more symptoms or greater impairment. The item scores in each domain are summated as the domain score. The total WOMAC score is the sum of the three domain scores, ranging from 0 to 96.^39^ For participants with completion of at least four of the five pain items, one of the two stiffness items, and 14 of the 17 function items, missing item values can be imputed with the average of the completed items in the relevant subscale.^40^ The Chinese version of WOMAC is valid, reliable, and sensitive in Chinese patients.^41^

#### Pain Rating Scale (PRS)

The PRS is a self- reported tool consisting of a scale with extreme anchors of ‘ no pain (0)’ to ‘ extreme pain (10)’^42^ to assess the severity of pain. The validity and reliability of the Chinese version is established.^43^

### Statistical analysis

Descriptive statistics were used to describe the patients’ sociodemographic information, lifestyle factors, disease characteristics, and PROs. One-way analysis of variance (ANOVA) was used to test the mean differences in the PROs between the groups of patients diagnosed with a back problem only, knee problem only, and both back and knee problems. Post hoc test (by Tukey’s honestly significant difference) was used to compare each group against the others. Pearson’s correlation between different PROs was examined. Multivariable regression was used to test the effect of disease characteristics, PRS score, and PEI-2 score on WOMAC total score, adjusted by sociodemographic characteristics.

Moderation analysis was used to test if enablement (PEI-2 score as the moderator) would interact with pain level (PRS score as the predictor) to influence HRQoL (WOMAC total score as the outcome). Moderation analysis hypothesises that a predictor and the interaction between a predictor and a moderator can predict the outcome if there is a ‘moderation effect’.^44^ The PRS and PEI-2 scores were standardised (converted to a *Z-*score) to avoid multicollinearity in the moderation analysis. Other variables that had significant effects on WOMAC total score were included as confounders in the moderation regression model. The moderation analysis was carried out by Hayes’s PROCESS macro^44^ for SPSS Model 1. In addition, a simple slope analysis was used to show the moderation effect.^45^ The mean of the estimated WOMAC total score (*Ŷ*) was calculated according to the following equation:


$$\hat{Y}=\beta_{1}\text{PEI}-2\;\text{score}+\beta_{2},\text{PRS}\;\text{score}+\beta_{3}(\text{PEI}-2\;\text{score}\times \text{PRS}\;\text{score})+\alpha$$

A 5% statistical significance was used for all analyses. All the analyses were carried out with IBM SPSS Statistics (version 27).

## RESULTS

### Sociodemographic, lifestyle, and disease characteristics

Table 1 shows the participants’ sociodemographic, lifestyle, and disease characteristics. Of the 1319 participants, 69.1% were female and the mean age was 68.80 years (standard deviation [SD] 10.16). The physician diagnoses were back problem only (22.4%), knee problem only (67.6%), and both back and knee problems (9.9%). The study population was similar in age and gender distribution to the back and knee problem patient population (mean age 67.28 years and 67.5% female) presenting to primary care clinics found in a territory-wide morbidity survey in 2021/2022 in Hong Kong.^46^ The mean and median GRS score was 2.45 (SD 0.64) and 2 (range 1 to 5), respectively.

**Table 1.** Baseline characteristics of participants (*N* = 1319)

**Characteristic**	***n***	**%**
**Sociodemographic**		
Sex		
Male	408	30.9
Female	911	69.1

Age, years		
18–50	41	3.1
51–60	203	15.4
61–70	510	38.7
71–80	363	27.5
>80	202	15.3

Education		
Primary or less	700	53.1
Secondary	509	38.6
Tertiary or above	107	8.1
Missing/unknown	3	0.2

Marital status		
Never married	86	6.5
Married	1004	76.1
Separated/divorced	55	4.2
Widowed	168	12.7
Missing/unknown	6	0.5

Occupation		
Unemployed/retired	710	53.8
Homemaker	309	23.4
Labour worker	138	10.5
Clerical worker	51	3.9
Professional or manager	34	2.6
Others	74	5.6
Missing/unknown	3	0.2

Household monthly income, HKD^a^		
0–$9999	670	50.8
$10 000–19 999	165	12.5
$20 000–29 999	83	6.3
>$29 999	120	9.1
Missing/unknown	281	21.3

**Lifestyle**		
Smoking		
Non-smoker	1173	88.9
Ex-smoker	91	6.9
Current smoker	50	3.8
Missing/unknown	5	0.4
Alcohol drinking		
Non-drinker	1136	86.1
Ex-drinker	50	3.8
Current drinker^b^	129	9.8
Missing/unknown	4	0.3

**Disease**		
Diagnosis of musculoskeletal problem		
Back only	296	22.4
Knee only	892	67.6
Both	131	9.9

Duration, years		
<1	216	16.4
1–5	439	33.3
6–10	251	19.0
>10	394	29.9
Missing/unknown	19	1.4

Total number of comorbidities		
0	128	9.7
1	628	47.6
2	372	28.2
3	154	11.7
≥4	37	2.8

Comorbidities		
No chronic disease	128	9.7
Heart disease	113	8.6
Hypertension	974	73.8
Stroke	40	3.0
Diabetes	348	26.4
Lung disease	27	2.0
Mental illness	57	4.3
Kidney disease	18	1.4
Other joint problem	185	14.0
Cancer	27	2.0
Other diseases	202	15.3

Doctor-reported GRS score		
1 (no problem)	28	2.1
2 (mild)	746	56.6
3 (moderate)	462	35.0
4 (severe)	67	5.1
5 (very severe)	5	0.4
Missing/unknown	11	0.8

a *Hong Kong population median household income, HKD 27 320 Hong Kong General Household Survey 2021.^47^*

b *Current drinker included participants who seldom drink to always drink alcohol. GRS = global rating scale on disease severity. HKD = Hong Kong Dollar.*

### PROs

Valid WOMAC scores (the primary outcome) were available from 1306 participants whose characteristics were similar to those with incomplete data (*n* = 13) (see Supplementary Table S1). Valid PEI-2 scores and PRS scores were available for 1258 and 1307 participants, respectively. Table 2 shows the overall mean WOMAC total score (20.93, SD 14.77), PEI-2 score (21.58, SD 3.49), PRS score (5.44, SD 2.32), and the distribution of these PRO scores by musculoskeletal diagnosis groups.

**Table 2.** Patient-reported outcomes by musculoskeletal problem diagnosis

**Measure**	**Overall, mean (SD) (*n* = 1306)**	**Back, mean (SD) (*n* = 296)**	**Knee, mean (SD) (*n* = 892)**	**Back and knee, mean (SD) (*n* = 131)**	***P*-value^a^**	**Post hoc test, group comparison^b^**
**WOMAC (*n* = 1306)**						
Pain (range 0 to 20)	5.17 (2.48)	5.35 (3.55)	4.96 (3.43)	6.20 (3.45)	<0.001	1 = 2, 1 = 3, 3>2
Stiffness (range 0 to 8)	1.65 (1.68)	1.70 (1.71)	1.58 (1.68)	1.95 (1.56)	0.057	1 = 2, 1 = 3, 2 = 3
Physical function (range 0 to 68)	14.15 (11.06)	13.73 (10.89)	13.84 (11.15)	17.11 (10.47)	0.005	1 = 2, 3>1, 3>2
Total score (range 0 to 96)	20.93 (14.77)	20.83 (14.52)	20.36 (14.93)	25.16 (13.58)	0.003	1 = 2, 3>1, 3>2

**PEI-2 score (*n* = 1258) (range 6 to 30)**	21.58 (3.49)	21.45 (3.55)	21.74 (3.46)	20.85 (3.46)	0.021	1 = 2, 1 = 3, 3>2

**PRS score (*n* = 1307) (range 0 to 10)**	5.44 (2.32)	5.73 (2.33)	5.28 (2.33)	5.91 (2.18)	0.001	1>2, 1 = 3, 3>2

a *One-way ANOVA was used for the analysis.*

b *For the post hoc test, 1 = back problem only group, 2 = knee problem only group, and 3 = both back and knee problem group. The difference in mean WOMAC total score was not significant between the back only and knee only groups (1 = 2), but was significantly higher in both the back and knee problem group (3) than those of the back problem only group (3>1) and knee problem only group (3>2). PEI-2 = Patient Enablement Instrument (higher score indicates more enabled). PRS = Pain Rating Scale (higher score indicates more pain). SD = standard deviation. WOMAC = Western Ontario and McMaster Universities Osteoarthritis Index (higher score indicates more limitation).*

One-way ANOVA analysis showed significant differences in WOMAC total, pain domain and physical function domain scores, PEI-2 score, and PRS score among diagnostic groups. Post hoc analysis (see Supplementary Table S2) found that participants with both back and knee problems had significantly higher WOMAC scores than participants with back or knee problems only. Participants with back problems only had significantly higher PRS scores than those with knee problems only.

Pearson correlation analysis (Table 3) found the WOMAC total score was positively correlated with the PRS score (*r* = 0.506, *P*<0.001) but negatively correlated with the PEI-2 score (*r* = −0.392, *P*<0.001). The PEI-2 score was negatively correlated with the PRS score (*r* = −0.202, *P*<0.001).

**Table 3.** Correlations between patient-reported outcomes

**Measure**	**Mean**	**SD**	**Pearson correlation coefficient, *r* ( *P*-value)**		
			**WOMAC total score**	**PEI-2 score**	**PRS score**
WOMAC total score (*n* = 1306)	20.93	14.77	—	−0.392 (<0.001)	0.506 (<0.001)
PEI-2 score (*n* = 1258)	21.58	3.49	−0.392 (<0.001)	—	−0.202 (<0.001)
PRS score (*n* = 1307)	5.44	2.32	0.506 (<0.001)	−0.202 (<0.001)	—

*PEI-2 = Patient Enablement Instrument (higher score indicates more enabled). PRS = Pain Rating Scale (higher score indicates more pain). SD = standard deviation. WOMAC = Western Ontario and McMaster Universities Osteoarthritis Index (higher score indicates more limitation).*

Multivariable regression analysis with adjustment for sociodemographic and lifestyle factors (Table 4) showed that the PRS score (β = 0.326, *P*<0.001), GRS score (β = 0.204, *P*<0.001), and number of comorbidities (β = 0.134, *P*<0.001) were positively associated with the WOMAC total score. A higher PEI-2 score was associated with a lower WOMAC total score (β = −0.260, *P*<0.001).

**Table 4.** Effect of disease characteristics, PRS, and PEI-2 on WOMAC total score (*N* = 1306)

**Variables**	**WOMAC total score**			

	**Unadjusted**		**Adjusted**	

	**Standardised** β**-coefficient**	***P*-value^a^**	**Standardised** β**-coefficient**	***P*-value^b^**
**Disease characteristics**				
Diagnosis of musculoskeletal disease				
Knee only^c^	Reference	Reference	Reference	—
Back only	−0.028	0.226	−0.022	0.409
Back and knee	0.035	0.124	0.041	0.117

Duration of musculoskeletal disease, years				
<1^c^	Reference	Reference	Reference	—
1–5	−0.030	0.342	−0.058	0.107
6–10	−0.014	0.633	−0.044	0.199
>10	0.031	0.326	−0.003	0.928

Total number of comorbidities	0.129	<0.001	0.134	<0.001

Doctor-reported GRS score	0.217	<0.001	0.204	<0.001

**PRS score**	0.372	<0.001	0.326	<0.001

**PEI-2 score**	−0.271	<0.001	−0.260	<0.001

a *Multivariable linear regression was used for the analysis.*

b *By multivariable linear regression adjusted for sociodemographic and lifestyle characteristics variables (including gender, age, education, marital status, occupation, household monthly income, drinking habit, and smoking habit).*

c *Reference category. GRS = global rating scale on disease severity. PEI-2 = Patient Enablement Instrument (higher score indicates more enabled). PRS = Pain Rating Scale (higher score indicates more pain). WOMAC = Western Ontario and McMaster Universities Osteoarthritis Index (higher score indicates more limitation).*

### Moderation analysis

The result of the moderation analysis is shown in Table 5. The effect of the interaction between PRS and PEI-2 (PRS × PEI-2) scores on the WOMAC total score was significant (β = −0.191, 95% confidence interval [CI] = −0.271 to −0.111]), suggesting that patient enablement weakened the effect of pain measured by PRS on the HRQoL measured by WOMAC. A sensitivity analysis was carried out by clusters (three clinics in each), which showed similar results (see Supplementary Table S3).

**Table 5.** Moderation of PEI-2 on the effect of PRS on WOMAC total score (*N* = 1221)^a^

**Variable**	**Standardised** β**-coefficient**	**95% CI**	**SE**	***t*-value**	***P*-value^b^**
PRS score	2.401	2.101 to 2.701	0.153	15.713	<0.001
PEI-2 score	−1.178	−1.367 to −0.988	0.097	−12.170	<0.001
PRS score × PEI-2 score	−0.191	−0.271 to −0.111	0.041	−4.704	<0.001

a *Only participants with valid data in all three patient-reported outcomes were included in the moderation analysis (*N *= 1221).*

b *Moderated regression analysis adjusted by gender, total number of comorbidities, physician global rating scale score on severity, and drinking status. PEI-2 = Patient Enablement Instrument (higher score indicates more enabled). PRS = Pain Rating Scale (higher score indicates more pain). SE = standard error. WOMAC = Western Ontario and McMaster Universities Osteoarthritis Index (higher score indicates more limitation).*

**Figure 1.**
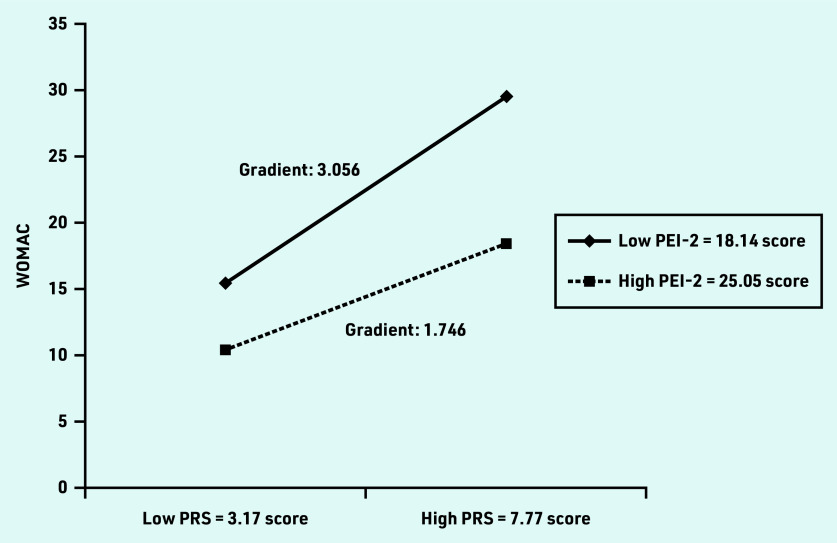
*Interaction of PRS and PEI-2 on predicting WOMAC total score. Effect of the PRS on the WOMAC total score in the low PEI-2 score (−1 SD) group — simple slope gradient 3.056 (95% CI = 2.626 to 3.486), SE 0.219,* t*-value = 13.941,* P*<0.001. Effect of the PRS on the WOMAC total score in the high PEI-2 (+1 SD) group — simple slope gradient 1.746 (95% CI = 1.367 to 2.126), SE 0.193,* t*-value = 9.030,* P*<0.001. PEI-2 = Patient Enablement Instrument (higher score indicates more enabled). PRS = Pain Rating Scale (higher score indicates more pain). SD = standard deviation. SE = standard error. WOMAC = Western Ontario and McMaster Universities Osteoarthritis Index (higher score indicates more limitation).*

Simple slope analysis (Figure 1) shows a different gradient effect of PRS score on WOMAC total score for participants with high (PEI-2 score 25.05) and low (PEI-2 score 18.14) levels of enablement. The relationship between WOMAC and PRS score was stronger for participants with a low level of PEI-2 (gradient 3.056 [95% CI = 2.626 to 3.486]) than for those with a high level of PEI-2 (gradient = 1.746 [95% CI = 1.367 to 2.126]). The difference in the gradient of the WOMAC total score plot from the low level of pain (PRS score 3.17) to a high level of pain (PRS 7.77) was greater (difference 1.31) at the low PEI-2 than the high PEI-2 level.

As the WOMAC total score includes the pain domain that may bias the association with PRS, a sensitivity analysis was carried out on the moderation effect using the WOMAC stiffness domain and physical function domain summation score (excluding the pain domain score) as the outcome. It showed similar results (see Supplementary Table S4 and Supplementary Figure S3), further substantiating that PEI-2 was a moderator between PRS and WOMAC total scores.

## DISCUSSION

### Summary

This study examined the moderation of patient enablement on the negative impact of pain on HRQoL in the context of chronic back and knee problems. The results confirmed that patient self-reported pain and enablement were the most significant determinants of HRQoL. As hypothesised, a higher level of pain was associated with more impairment, and a higher level of enablement was associated with less impairment of HRQoL. This study has established that enablement was a significant moderator of the association between pain and HRQoL. Specifically, a high level of enablement weakens the effect of pain on HRQoL impairment.

### Strengths and limitations

The strength of the study was the large number of participants recruited from multiple primary care clinics. The age and gender distribution of the participants was similar to that of the back and knee problem patient population found in the Hong Kong territory-wide primary care morbidity survey in 2021/2022. The results may be generalisable to the general Hong Kong population. The PRO data were measured with standard valid measures and they established patient enablement as a moderator of the association between pain and HRQoL. The sensitivity analyses showed consistent results, supporting reliability. There were a few limitations to this study. First, the analysis of cross-sectional data could not ascertain a causal relationship, and the correlations between PEI and WOMAC (−0.39) and PRS (−0.20) scores were relatively small. Second, the results from patients in public primary care clinics may not be applicable to those managed in the private sector. Third, self-report data are subject to bias from recall and a tendency to give socially acceptable answers. The authors believe these biases applied to all participants and should not have affected the moderation analysis results. Finally, the results from Chinese patients may not be generalisable to populations who are not Chinese.

### Comparison with existing literature

The current study confirmed the findings from previous studies on the negative association between pain severity and HRQoL^8^^–^11^,^^27^^,^^48^^–^50 and the positive association between enablement and HRQoL.^51^ A significant, although weak, negative association was found between enablement and pain, which moderated the association between pain and HRQoL. Theoretically, enablement promotes the patient’s ability to control their health, life, and coping strategies, which can lessen pain, depression, and limitation in daily functioning; therefore, it can improve HRQoL.^26^^,^^27^^,^^52^ Another mechanism by which enablement helps is through improved health literacy.^25^ Studies in Australia have shown that patients with chronic diseases who were more enabled were more likely to have a higher level of health literacy and to actively manage their health.^20^ In addition, health literacy can influence an individual’s illness perception resulting in better coping ability.^53^

### Implications for practice

The findings on the role of enablement as a moderator between pain and HRQoL has implications for a paradigm change in clinical practice. Most chronic musculoskeletal problems are progressive degenerative conditions with no cure. Primary care physicians should recognise patient enablement as a first-line intervention for chronic musculoskeletal problems. The scope of patient-centred care should be broadened to include an assessment of HRQoL and enablement so that management can be customised to enhance coping, health literacy, and self-care to maximise functioning. A reorientation of the treatment goal may help reduce the use of analgesics that can cause serious side effects and are subject to misuse.^54^^,^^55^ Education about the nature of the musculoskeletal problem and self- care strategies should be part of routine care for these patients.^56^ Exercise and self-care management programmes can reduce pain and improve function in patients with musculoskeletal problems.^57^^,^^58^ Appropriate referrals to other healthcare professionals can further enable patients to overcome specific environmental limitations. For instance, occupational therapists can advise on home modification so that patients can continue to live comfortably at home.

In conclusion, patient enablement moderated the negative impact of pain on HRQoL. Higher level of enablement can lessen the impairment of HRQoL related to chronic back and knee pain. Education, exercise, and self-care programmes that aim to enhance patients’ health literacy, functioning, and ability to cope with their illness should become routine treatments for chronic musculoskeletal problems. Future studies with more diverse populations are required to establish the interaction between enablement and pain on HRQoL, and how patient enablement can be effectively enhanced in patients with chronic musculoskeletal problems.

## Data

The HKU-HAHKWC IRB does not approve open access to data collected from patients. The dataset can only be accessed by persons authorised by the principal investigator. Datasets can be made available to readers upon request with justification.
